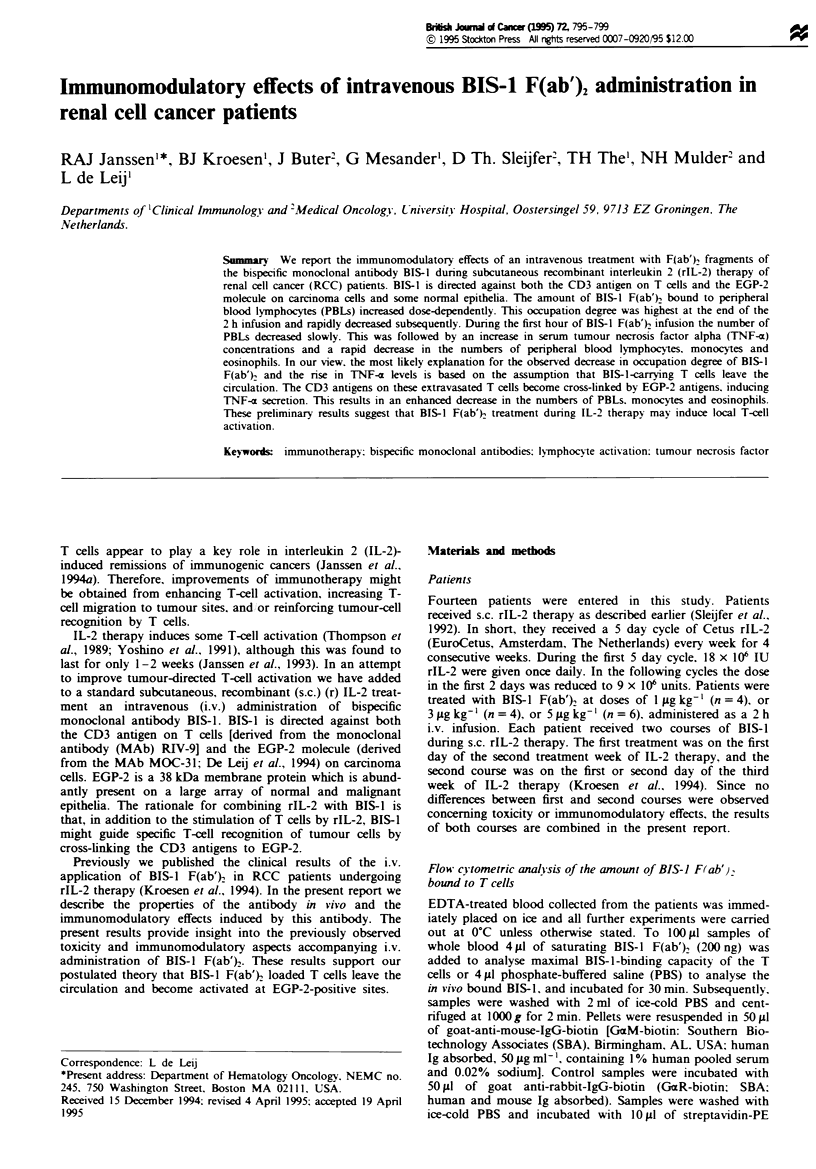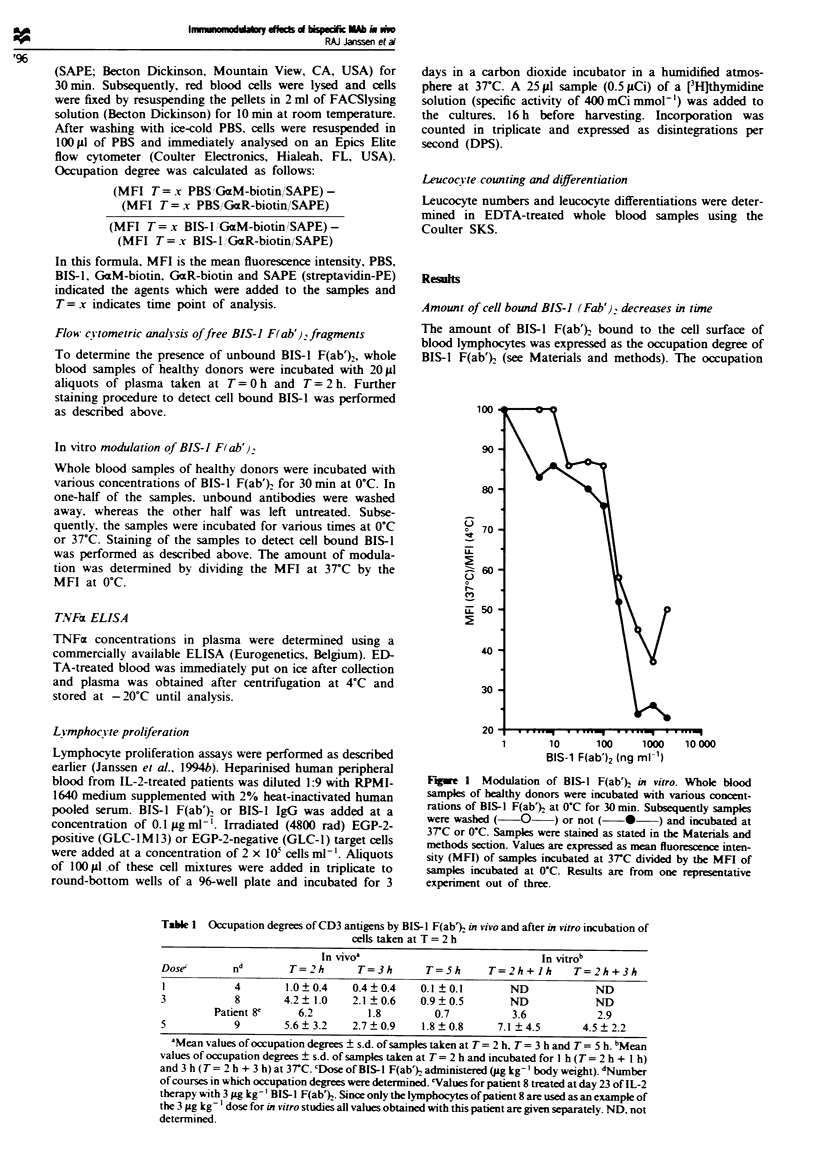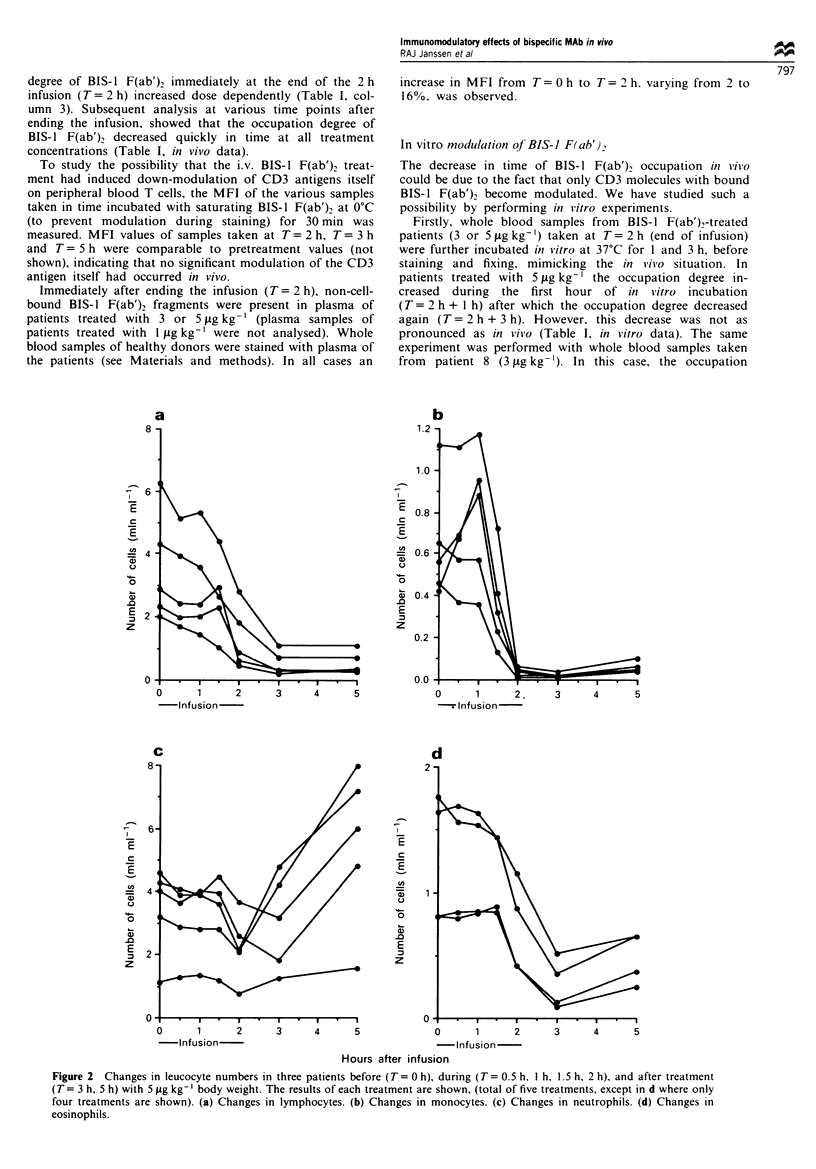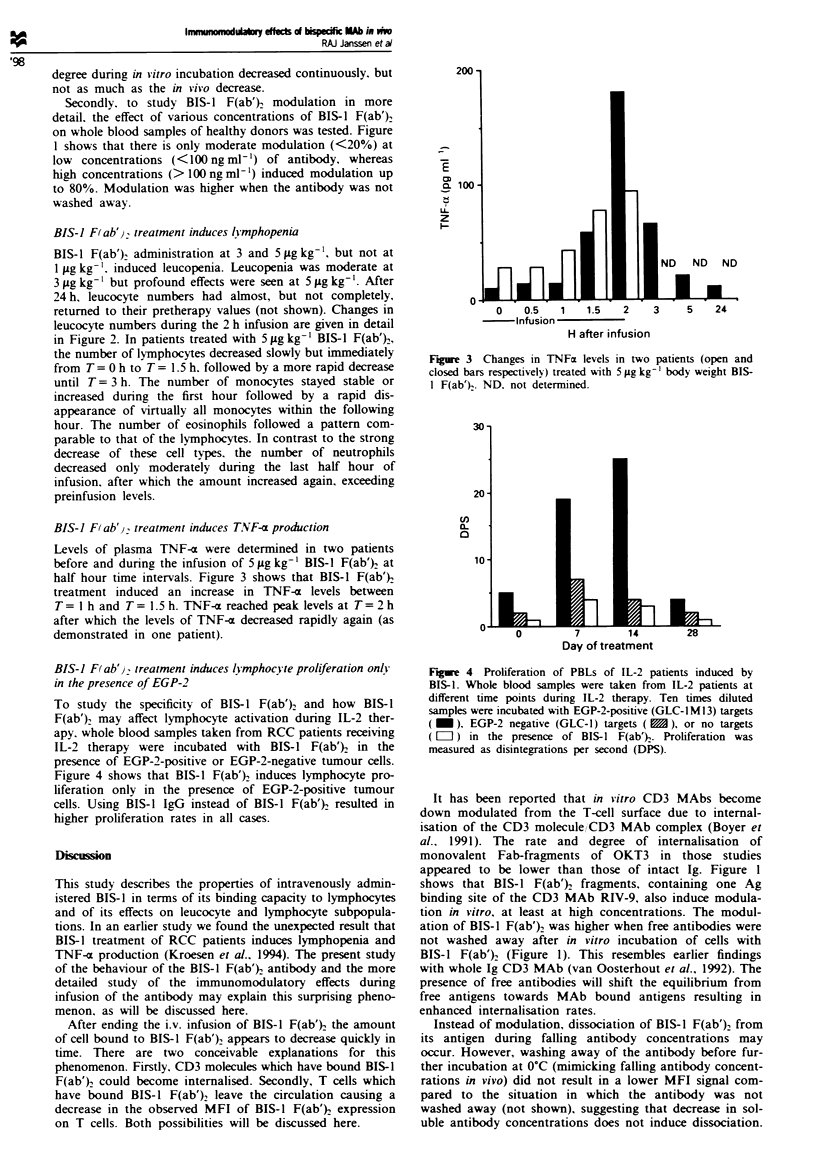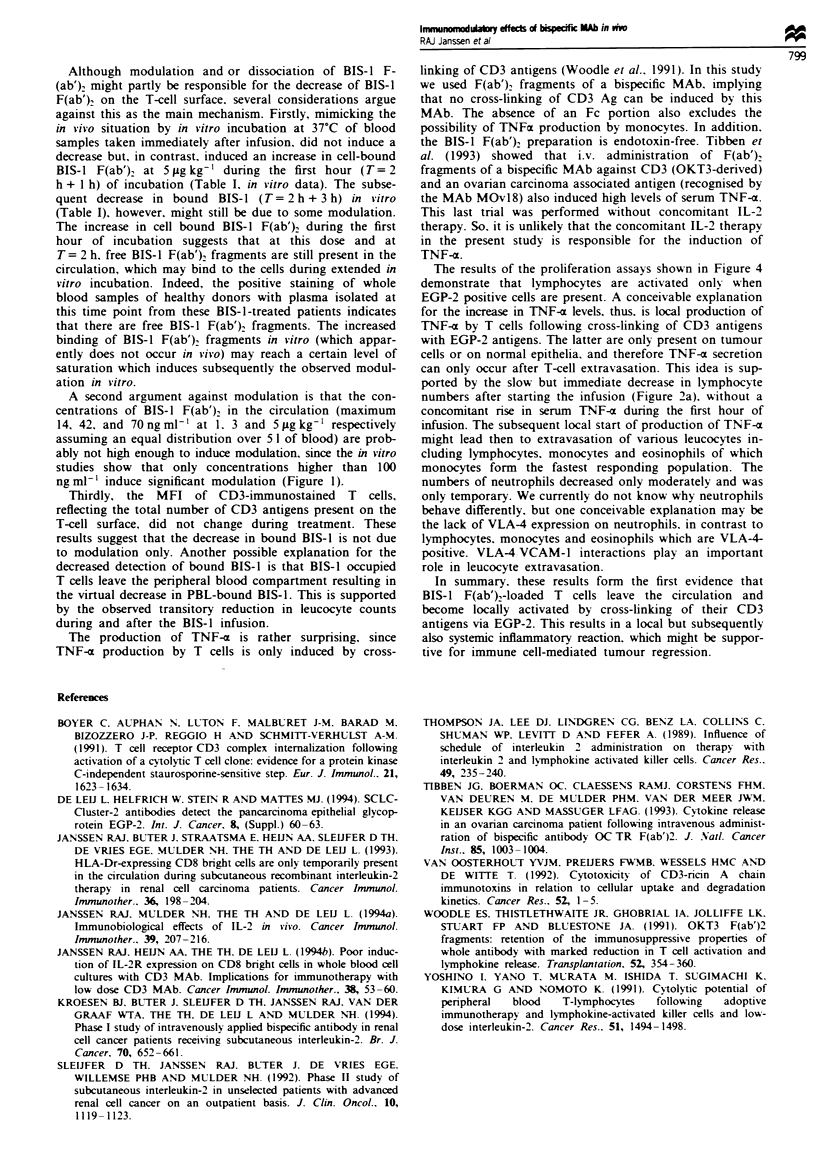# Immunomodulatory effects of intravenous BIS-1 F(ab')2 administration in renal cell cancer patients.

**DOI:** 10.1038/bjc.1995.414

**Published:** 1995-09

**Authors:** R. A. Janssen, B. J. Kroesen, J. Buter, G. Mesander, D. T. Sleijfer, T. H. The, N. H. Mulder, L. de Leij

**Affiliations:** Department of Clinical Immunology, University Hospital, Groningen, The Netherlands.

## Abstract

We report the immunomodulatory effects of an intravenous treatment with F(ab')2 fragments of the bispecific monoclonal antibody BIS-1 during subcutaneous recombinant interleukin 2 (rIL-2) therapy of renal cell cancer (RCC) patients. BIS-1 is directed against both the CD3 antigen on T cells and the EGP-2 molecule on carcinoma cells and some normal epithelia. The amount of BIS-1 F(ab')2 bound to peripheral blood lymphocytes (PBLs) increased dose-dependently. This occupation degree was highest at the end of the 2 h infusion and rapidly decreased subsequently. During the first hour of BIS-1 F(ab')2 infusion the number of PBLs decreased slowly. This was followed by an increase in serum tumour necrosis factor alpha (TNF-alpha) concentrations and a rapid decrease in the numbers of peripheral blood lymphocytes, monocytes and eosinophils. In our view, the most likely explanation for the observed decrease in occupation degree of BIS-1 F(ab')2 and the rise in TNF-alpha levels is based on the assumption that BIS-1-carrying T cells leave the circulation. The CD3 antigens on these extravasated T cells become cross-linked by EGP-2 antigens, inducing TNF-alpha secretion. This results in an enhanced decrease in the numbers of PBLs, monocytes and eosinophils. These preliminary results suggest that BIS-1 F(ab')2 treatment during IL-2 therapy may induce local T-cell activation.


British Journal of Cancer (1995) 72, 795–799

© 1995 Stockton Press All rights reserved 0007-0920/95 $12.00

### Immunomodulatory effects of intravenous BIS-1 F(ab')$_2$ administration in renal cell cancer patients

RAJ Janssen[1]*, BJ Kroesen[1], J Buter[2], G Mesander[1], D Th. Sleijfer[2], TH The[1], NH Mulder[2] and L de Leij[1]

*Departments of [1]Clinical Immunology and [2]Medical Oncology, University Hospital, Oostersingel 59, 9713 EZ Groningen, The Netherlands.*

**Summary** We report the immunomodulatory effects of an intravenous treatment with F(ab')$_2$ fragments of the bispecific monoclonal antibody BIS-1 during subcutaneous recombinant interleukin 2 (rIL-2) therapy of renal cell cancer (RCC) patients. BIS-1 is directed against both the CD3 antigen on T cells and the EGP-2 molecule on carcinoma cells and some normal epithelia. The amount of BIS-1 F(ab')$_2$ bound to peripheral blood lymphocytes (PBLs) increased dose-dependently. This occupation degree was highest at the end of the 2 h infusion and rapidly decreased subsequently. During the first hour of BIS-1 F(ab')$_2$ infusion the number of PBLs decreased slowly. This was followed by an increase in serum tumour necrosis factor alpha (TNF-$\alpha$) concentrations and a rapid decrease in the numbers of peripheral blood lymphocytes, monocytes and eosinophils. In our view, the most likely explanation for the observed decrease in occupation degree of BIS-1 F(ab')$_2$ and the rise in TNF-$\alpha$ levels is based on the assumption that BIS-1-carrying T cells leave the circulation. The CD3 antigens on these extravasated T cells become cross-linked by EGP-2 antigens, inducing TNF-$\alpha$ secretion. This results in an enhanced decrease in the numbers of PBLs, monocytes and eosinophils. These preliminary results suggest that BIS-1 F(ab')$_2$ treatment during IL-2 therapy may induce local T-cell activation.

**Keywords:** immunotherapy; bispecific monoclonal antibodies; lymphocyte activation; tumour necrosis factor

T cells appear to play a key role in interleukin 2 (IL-2)-induced remissions of immunogenic cancers (Janssen *et al.*, 1994*a*). Therefore, improvements of immunotherapy might be obtained from enhancing T-cell activation, increasing T-cell migration to tumour sites, and/or reinforcing tumour-cell recognition by T cells.

IL-2 therapy induces some T-cell activation (Thompson *et al.*, 1989; Yoshino *et al.*, 1991), although this was found to last for only 1–2 weeks (Janssen *et al.*, 1993). In an attempt to improve tumour-directed T-cell activation we have added to a standard subcutaneous, recombinant (s.c.) (r) IL-2 treatment an intravenous (i.v.) administration of bispecific monoclonal antibody BIS-1. BIS-1 is directed against both the CD3 antigen on T cells [derived from the monoclonal antibody (MAb) RIV-9] and the EGP-2 molecule (derived from the MAb MOC-31; De Leij *et al.*, 1994) on carcinoma cells. EGP-2 is a 38 kDa membrane protein which is abundantly present on a large array of normal and malignant epithelia. The rationale for combining rIL-2 with BIS-1 is that, in addition to the stimulation of T cells by rIL-2, BIS-1 might guide specific T-cell recognition of tumour cells by cross-linking the CD3 antigens to EGP-2.

Previously we published the clinical results of the i.v. application of BIS-1 F(ab')$_2$ in RCC patients undergoing rIL-2 therapy (Kroesen *et al.*, 1994). In the present report we describe the properties of the antibody *in vivo* and the immunomodulatory effects induced by this antibody. The present results provide insight into the previously observed toxicity and immunomodulatory aspects accompanying i.v. administration of BIS-1 F(ab')$_2$. These results support our postulated theory that BIS-1 F(ab')$_2$ loaded T cells leave the circulation and become activated at EGP-2-positive sites.

Correspondence: L de Leij
*Present address: Department of Hematology Oncology, NEMC no. 245, 750 Washington Street, Boston MA 02111, USA.
Received 15 December 1994; revised 4 April 1995; accepted 19 April 1995

### Materials and methods

### Patients

Fourteen patients were entered in this study. Patients received s.c. rIL-2 therapy as described earlier (Sleijfer *et al.*, 1992). In short, they received a 5 day cycle of Cetus rIL-2 (EuroCetus, Amsterdam, The Netherlands) every week for 4 consecutive weeks. During the first 5 day cycle, $18 \times 10^6$ IU rIL-2 were given once daily. In the following cycles the dose in the first 2 days was reduced to $9 \times 10^6$ units. Patients were treated with BIS-1 F(ab')$_2$ at doses of $1\ \mu g\ kg^{-1}$ ($n = 4$), or $3\ \mu g\ kg^{-1}$ ($n = 4$), or $5\ \mu g\ kg^{-1}$ ($n = 6$), administered as a 2 h i.v. infusion. Each patient received two courses of BIS-1 during s.c. rIL-2 therapy. The first treatment was on the first day of the second treatment week of IL-2 therapy, and the second course was on the first or second day of the third week of IL-2 therapy (Kroesen *et al.*, 1994). Since no differences between first and second courses were observed concerning toxicity or immunomodulatory effects, the results of both courses are combined in the present report.

### Flow cytometric analysis of the amount of BIS-1 F(ab')$_2$ bound to T cells

EDTA-treated blood collected from the patients was immediately placed on ice and all further experiments were carried out at 0°C unless otherwise stated. To 100 µl samples of whole blood 4 µl of saturating BIS-1 F(ab')$_2$ (200 ng) was added to analyse maximal BIS-1-binding capacity of the T cells or 4 µl phosphate-buffered saline (PBS) to analyse the *in vivo* bound BIS-1, and incubated for 30 min. Subsequently, samples were washed with 2 ml of ice-cold PBS and centrifuged at 1000 *g* for 2 min. Pellets were resuspended in 50 µl of goat-anti-mouse-IgG-biotin [GαM-biotin: Southern Biotechnology Associates (SBA), Birmingham, AL, USA; human Ig absorbed, $50\ \mu g\ ml^{-1}$, containing 1% human pooled serum and 0.02% sodium]. Control samples were incubated with 50 µl of goat anti-rabbit-IgG-biotin (GαR-biotin; SBA; human and mouse Ig absorbed). Samples were washed with ice-cold PBS and incubated with 10 µl of streptavidin-PE

(SAPE; Becton Dickinson, Mountain View, CA, USA) for 30 min. Subsequently, red blood cells were lysed and cells were fixed by resuspending the pellets in 2 ml of FACSlysing solution (Becton Dickinson) for 10 min at room temperature. After washing with ice-cold PBS, cells were resuspended in 100 μl of PBS and immediately analysed on an Epics Elite flow cytometer (Coulter Electronics, Hialeah, FL, USA). Occupation degree was calculated as follows:

$$\frac{(\text{MFI } T = x \text{ PBS/G}\alpha\text{M-biotin/SAPE}) - (\text{MFI } T = x \text{ PBS/G}\alpha\text{R-biotin/SAPE})}{(\text{MFI } T = x \text{ BIS-1/G}\alpha\text{M-biotin/SAPE}) - (\text{MFI } T = x \text{ BIS-1/G}\alpha\text{R-biotin/SAPE})}$$

In this formula, MFI is the mean fluorescence intensity, PBS, BIS-1, GαM-biotin, GαR-biotin and SAPE (streptavidin-PE) indicated the agents which were added to the samples and $T = x$ indicates time point of analysis.

*Flow cytometric analysis of free BIS-1 F(ab')$_2$ fragments*

To determine the presence of unbound BIS-1 F(ab')$_2$, whole blood samples of healthy donors were incubated with 20 μl aliquots of plasma taken at $T = 0$ h and $T = 2$ h. Further staining procedure to detect cell bound BIS-1 was performed as described above.

In vitro *modulation of BIS-1 F(ab')$_2$*

Whole blood samples of healthy donors were incubated with various concentrations of BIS-1 F(ab')$_2$ for 30 min at 0°C. In one-half of the samples, unbound antibodies were washed away, whereas the other half was left untreated. Subsequently, the samples were incubated for various times at 0°C or 37°C. Staining of the samples to detect cell bound BIS-1 was performed as described above. The amount of modulation was determined by dividing the MFI at 37°C by the MFI at 0°C.

*TNFα ELISA*

TNFα concentrations in plasma were determined using a commercially available ELISA (Eurogenetics, Belgium). EDTA-treated blood was immediately put on ice after collection and plasma was obtained after centrifugation at 4°C and stored at −20°C until analysis.

*Lymphocyte proliferation*

Lymphocyte proliferation assays were performed as described earlier (Janssen *et al.*, 1994*b*). Heparinised human peripheral blood from IL-2-treated patients was diluted 1:9 with RPMI-1640 medium supplemented with 2% heat-inactivated human pooled serum. BIS-1 F(ab')$_2$ or BIS-1 IgG was added at a concentration of 0.1 μg ml$^{-1}$. Irradiated (4800 rad) EGP-2-positive (GLC-1M13) or EGP-2-negative (GLC-1) target cells were added at a concentration of $2 \times 10^5$ cells ml$^{-1}$. Aliquots of 100 μl of these cell mixtures were added in triplicate to round-bottom wells of a 96-well plate and incubated for 3 days in a carbon dioxide incubator in a humidified atmosphere at 37°C. A 25 μl sample (0.5 μCi) of a [$^3$H]thymidine solution (specific activity of 400 mCi mmol$^{-1}$) was added to the cultures, 16 h before harvesting. Incorporation was counted in triplicate and expressed as disintegrations per second (DPS).

*Leucocyte counting and differentiation*

Leucocyte numbers and leucocyte differentiations were determined in EDTA-treated whole blood samples using the Coulter SKS.

### Results

*Amount of cell bound BIS-1 (Fab')$_2$ decreases in time*

The amount of BIS-1 F(ab')$_2$ bound to the cell surface of blood lymphocytes was expressed as the occupation degree of BIS-1 F(ab')$_2$ (see Materials and methods). The occupation

**Figure 1** Modulation of BIS-1 F(ab')$_2$ *in vitro*. Whole blood samples of healthy donors were incubated with various concentrations of BIS-1 F(ab')$_2$ at 0°C for 30 min. Subsequently samples were washed (——O——) or not (——●——) and incubated at 37°C or 0°C. Samples were stained as stated in the Materials and methods section. Values are expressed as mean fluorescence intensity (MFI) of samples incubated at 37°C divided by the MFI of samples incubated at 0°C. Results are from one representative experiment out of three.

**Table 1** Occupation degrees of CD3 antigens by BIS-1 F(ab')$_2$ *in vivo* and after *in vitro* incubation of cells taken at T = 2 h

| | | In vivo[a] | | | In vitro[b] | |
|---|---|---|---|---|---|---|
| *Dose*[c] | n[d] | $T = 2\,h$ | $T = 3\,h$ | $T = 5\,h$ | $T = 2\,h + 1\,h$ | $T = 2\,h + 3\,h$ |
| 1 | 4 | 1.0 ± 0.4 | 0.4 ± 0.4 | 0.1 ± 0.1 | ND | ND |
| 3 | 8 | 4.2 ± 1.0 | 2.1 ± 0.6 | 0.9 ± 0.5 | ND | ND |
| | Patient 8[e] | 6.2 | 1.8 | 0.7 | 3.6 | 2.9 |
| 5 | 9 | 5.6 ± 3.2 | 2.7 ± 0.9 | 1.8 ± 0.8 | 7.1 ± 4.5 | 4.5 ± 2.2 |

[a]Mean values of occupation degrees ± s.d. of samples taken at $T = 2$ h, $T = 3$ h and $T = 5$ h. [b]Mean values of occupation degrees ± s.d. of samples taken at $T = 2$ h and incubated for 1 h ($T = 2$ h + 1 h) and 3 h ($T = 2$ h + 3 h) at 37°C. [c]Dose of BIS-1 F(ab')$_2$ administered (μg kg$^{-1}$ body weight). [d]Number of courses in which occupation degrees were determined. [e]Values for patient 8 treated at day 23 of IL-2 therapy with 3 μg kg$^{-1}$ BIS-1 F(ab')$_2$. Since only the lymphocytes of patient 8 are used as an example of the 3 μg kg$^{-1}$ dose for *in vitro* studies all values obtained with this patient are given separately. ND, not determined.

degree of BIS-1 $F(ab')_2$ immediately at the end of the 2 h infusion ($T = 2$ h) increased dose dependently (Table I, column 3). Subsequent analysis at various time points after ending the infusion, showed that the occupation degree of BIS-1 $F(ab')_2$ decreased quickly in time at all treatment concentrations (Table I, *in vivo* data).

To study the possibility that the i.v. BIS-1 $F(ab')_2$ treatment had induced down-modulation of CD3 antigens itself on peripheral blood T cells, the MFI of the various samples taken in time incubated with saturating BIS-1 $F(ab')_2$ at 0°C (to prevent modulation during staining) for 30 min was measured. MFI values of samples taken at $T = 2$ h, $T = 3$ h and $T = 5$ h were comparable to pretreatment values (not shown), indicating that no significant modulation of the CD3 antigen itself had occurred *in vivo*.

Immediately after ending the infusion ($T = 2$ h), non-cell-bound BIS-1 $F(ab')_2$ fragments were present in plasma of patients treated with 3 or 5 $\mu g\ kg^{-1}$ (plasma samples of patients treated with 1 $\mu g\ kg^{-1}$ were not analysed). Whole blood samples of healthy donors were stained with plasma of the patients (see Materials and methods). In all cases an increase in MFI from $T = 0$ h to $T = 2$ h, varying from 2 to 16%, was observed.

### In vitro *modulation of BIS-1 $F(ab')_2$*

The decrease in time of BIS-1 $F(ab')_2$ occupation *in vivo* could be due to the fact that only CD3 molecules with bound BIS-1 $F(ab')_2$ become modulated. We have studied such a possibility by performing *in vitro* experiments.

Firstly, whole blood samples from BIS-1 $F(ab')_2$-treated patients (3 or 5 $\mu g\ kg^{-1}$) taken at $T = 2$ h (end of infusion) were further incubated *in vitro* at 37°C for 1 and 3 h, before staining and fixing, mimicking the *in vivo* situation. In patients treated with 5 $\mu g\ kg^{-1}$ the occupation degree increased during the first hour of *in vitro* incubation ($T = 2$ h + 1 h) after which the occupation degree decreased again ($T = 2$ h + 3 h). However, this decrease was not as pronounced as *in vivo* (Table I, *in vitro* data). The same experiment was performed with whole blood samples taken from patient 8 (3 $\mu g\ kg^{-1}$). In this case, the occupation

**Figure 2** Changes in leucocyte numbers in three patients before ($T = 0$ h), during ($T = 0.5$ h, 1 h, 1.5 h, 2 h), and after treatment ($T = 3$ h, 5 h) with 5 $\mu g\ kg^{-1}$ body weight. The results of each treatment are shown, (total of five treatments, except in **d** where only four treatments are shown). (**a**) Changes in lymphocytes. (**b**) Changes in monocytes. (**c**) Changes in neutrophils. (**d**) Changes in eosinophils.

degree during *in vitro* incubation decreased continuously, but not as much as the *in vivo* decrease.

Secondly, to study BIS-1 F(ab')$_2$ modulation in more detail, the effect of various concentrations of BIS-1 F(ab')$_2$ on whole blood samples of healthy donors was tested. Figure 1 shows that there is only moderate modulation (<20%) at low concentrations (<100 ng ml$^{-1}$) of antibody, whereas high concentrations (>100 ng ml$^{-1}$) induced modulation up to 80%. Modulation was higher when the antibody was not washed away.

### *BIS-1 F(ab')$_2$ treatment induces lymphopenia*

BIS-1 F(ab')$_2$ administration at 3 and 5 μg kg$^{-1}$, but not at 1 μg kg$^{-1}$, induced leucopenia. Leucopenia was moderate at 3 μg kg$^{-1}$ but profound effects were seen at 5 μg kg$^{-1}$. After 24 h, leucocyte numbers had almost, but not completely, returned to their pretherapy values (not shown). Changes in leucocyte numbers during the 2 h infusion are given in detail in Figure 2. In patients treated with 5 μg kg$^{-1}$ BIS-1 F(ab')$_2$, the number of lymphocytes decreased slowly but immediately from $T = 0$ h to $T = 1.5$ h, followed by a more rapid decrease until $T = 3$ h. The number of monocytes stayed stable or increased during the first hour followed by a rapid disappearance of virtually all monocytes within the following hour. The number of eosinophils followed a pattern comparable to that of the lymphocytes. In contrast to the strong decrease of these cell types, the number of neutrophils decreased only moderately during the last half hour of infusion, after which the amount increased again, exceeding preinfusion levels.

### *BIS-1 F(ab')$_2$ treatment induces TNF-α production*

Levels of plasma TNF-α were determined in two patients before and during the infusion of 5 μg kg$^{-1}$ BIS-1 F(ab')$_2$ at half hour time intervals. Figure 3 shows that BIS-1 F(ab')$_2$ treatment induced an increase in TNF-α levels between $T = 1$ h and $T = 1.5$ h. TNF-α reached peak levels at $T = 2$ h after which the levels of TNF-α decreased rapidly again (as demonstrated in one patient).

### *BIS-1 F(ab')$_2$ treatment induces lymphocyte proliferation only in the presence of EGP-2*

To study the specificity of BIS-1 F(ab')$_2$ and how BIS-1 F(ab')$_2$ may affect lymphocyte activation during IL-2 therapy, whole blood samples taken from RCC patients receiving IL-2 therapy were incubated with BIS-1 F(ab')$_2$ in the presence of EGP-2-positive or EGP-2-negative tumour cells. Figure 4 shows that BIS-1 F(ab')$_2$ induces lymphocyte proliferation only in the presence of EGP-2-positive tumour cells. Using BIS-1 IgG instead of BIS-1 F(ab')$_2$ resulted in higher proliferation rates in all cases.

### Discussion

This study describes the properties of intravenously administered BIS-1 in terms of its binding capacity to lymphocytes and of its effects on leucocyte and lymphocyte subpopulations. In an earlier study we found the unexpected result that BIS-1 treatment of RCC patients induces lymphopenia and TNF-α production (Kroesen *et al*., 1994). The present study of the behaviour of the BIS-1 F(ab')$_2$ antibody and the more detailed study of the immunomodulatory effects during infusion of the antibody may explain this surprising phenomenon, as will be discussed here.

After ending the i.v. infusion of BIS-1 F(ab')$_2$ the amount of cell bound to BIS-1 F(ab')$_2$ appears to decrease quickly in time. There are two conceivable explanations for this phenomenon. Firstly, CD3 molecules which have bound BIS-1 F(ab')$_2$ could become internalised. Secondly, T cells which have bound BIS-1 F(ab')$_2$ leave the circulation causing a decrease in the observed MFI of BIS-1 F(ab')$_2$ expression on T cells. Both possibilities will be discussed here.

**Figure 3** Changes in TNFα levels in two patients (open and closed bars respectively) treated with 5 μg kg$^{-1}$ body weight BIS-1 F(ab')$_2$. ND, not determined.

**Figure 4** Proliferation of PBLs of IL-2 patients induced by BIS-1. Whole blood samples were taken from IL-2 patients at different time points during IL-2 therapy. Ten times diluted samples were incubated with EGP-2-positive (GLC-1M13) targets (■), EGP-2 negative (GLC-1) targets (▨), or no targets (□) in the presence of BIS-1 F(ab')$_2$. Proliferation was measured as disintegrations per second (DPS).

It has been reported that *in vitro* CD3 MAbs become down modulated from the T-cell surface due to internalisation of the CD3 molecule/CD3 MAb complex (Boyer *et al*., 1991). The rate and degree of internalisation of monovalent Fab-fragments of OKT3 in those studies appeared to be lower than those of intact Ig. Figure 1 shows that BIS-1 F(ab')$_2$ fragments, containing one Ag binding site of the CD3 MAb RIV-9, also induce modulation *in vitro*, at least at high concentrations. The modulation of BIS-1 F(ab')$_2$ was higher when free antibodies were not washed away after *in vitro* incubation of cells with BIS-1 F(ab')$_2$ (Figure 1). This resembles earlier findings with whole Ig CD3 MAb (van Oosterhout *et al*., 1992). The presence of free antibodies will shift the equilibrium from free antigens towards MAb bound antigens resulting in enhanced internalisation rates.

Instead of modulation, dissociation of BIS-1 F(ab')$_2$ from its antigen during falling antibody concentrations may occur. However, washing away of the antibody before further incubation at 0°C (mimicking falling antibody concentrations *in vivo*) did not result in a lower MFI signal compared to the situation in which the antibody was not washed away (not shown), suggesting that decrease in soluble antibody concentrations does not induce dissociation.

Although modulation and or dissociation of BIS-1 F-(ab')$_2$ might partly be responsible for the decrease of BIS-1 F(ab')$_2$ on the T-cell surface, several considerations argue against this as the main mechanism. Firstly, mimicking the *in vivo* situation by *in vitro* incubation at 37°C of blood samples taken immediately after infusion, did not induce a decrease but, in contrast, induced an increase in cell-bound BIS-1 F(ab')$_2$ at $5\,\mu g\,kg^{-1}$ during the first hour ($T = 2$ h + 1 h) of incubation (Table I, *in vitro* data). The subsequent decrease in bound BIS-1 ($T = 2\,h + 3\,h$) *in vitro* (Table I), however, might still be due to some modulation. The increase in cell bound BIS-1 F(ab')$_2$ during the first hour of incubation suggests that at this dose and at $T = 2$ h, free BIS-1 F(ab')$_2$ fragments are still present in the circulation, which may bind to the cells during extended *in vitro* incubation. Indeed, the positive staining of whole blood samples of healthy donors with plasma isolated at this time point from these BIS-1-treated patients indicates that there are free BIS-1 F(ab')$_2$ fragments. The increased binding of BIS-1 F(ab')$_2$ fragments *in vitro* (which apparently does not occur *in vivo*) may reach a certain level of saturation which induces subsequently the observed modulation *in vitro*.

A second argument against modulation is that the concentrations of BIS-1 F(ab')$_2$ in the circulation (maximum 14, 42, and $70\,ng\,ml^{-1}$ at 1, 3 and $5\,\mu g\,kg^{-1}$ respectively assuming an equal distribution over 5 l of blood) are probably not high enough to induce modulation, since the *in vitro* studies show that only concentrations higher than 100 $ng\,ml^{-1}$ induce significant modulation (Figure 1).

Thirdly, the MFI of CD3-immunostained T cells, reflecting the total number of CD3 antigens present on the T-cell surface, did not change during treatment. These results suggest that the decrease in bound BIS-1 is not due to modulation only. Another possible explanation for the decreased detection of bound BIS-1 is that BIS-1 occupied T cells leave the peripheral blood compartment resulting in the virtual decrease in PBL-bound BIS-1. This is supported by the observed transitory reduction in leucocyte counts during and after the BIS-1 infusion.

The production of TNF-α is rather surprising, since TNF-α production by T cells is only induced by cross-linking of CD3 antigens (Woodle *et al*., 1991). In this study we used F(ab')$_2$ fragments of a bispecific MAb, implying that no cross-linking of CD3 Ag can be induced by this MAb. The absence of an Fc portion also excludes the possibility of TNFα production by monocytes. In addition, the BIS-1 F(ab')$_2$ preparation is endotoxin-free. Tibben *et al*. (1993) showed that i.v. administration of F(ab')$_2$ fragments of a bispecific MAb against CD3 (OKT3-derived) and an ovarian carcinoma associated antigen (recognised by the MAb MOv18) also induced high levels of serum TNF-α. This last trial was performed without concomitant IL-2 therapy. So, it is unlikely that the concomitant IL-2 therapy in the present study is responsible for the induction of TNF-α.

The results of the proliferation assays shown in Figure 4 demonstrate that lymphocytes are activated only when EGP-2 positive cells are present. A conceivable explanation for the increase in TNF-α levels, thus, is local production of TNF-α by T cells following cross-linking of CD3 antigens with EGP-2 antigens. The latter are only present on tumour cells or on normal epithelia, and therefore TNF-α secretion can only occur after T-cell extravasation. This idea is supported by the slow but immediate decrease in lymphocyte numbers after starting the infusion (Figure 2a), without a concomitant rise in serum TNF-α during the first hour of infusion. The subsequent local start of production of TNF-α might lead then to extravasation of various leucocytes including lymphocytes, monocytes and eosinophils of which monocytes form the fastest responding population. The numbers of neutrophils decreased only moderately and was only temporary. We currently do not know why neutrophils behave differently, but one conceivable explanation may be the lack of VLA-4 expression on neutrophils, in contrast to lymphocytes, monocytes and eosinophils which are VLA-4-positive. VLA-4/VCAM-1 interactions play an important role in leucocyte extravasation.

In summary, these results form the first evidence that BIS-1 F(ab')$_2$-loaded T cells leave the circulation and become locally activated by cross-linking of their CD3 antigens via EGP-2. This results in a local but subsequently also systemic inflammatory reaction, which might be supportive for immune cell-mediated tumour regression.

### References

BOYER C, AUPHAN N, LUTON F, MALBURET J-M, BARAD M, BIZOZZERO J-P, REGGIO H AND SCHMITT-VERHULST A-M. (1991). T cell receptor CD3 complex internalization following activation of a cytolytic T cell clone: evidence for a protein kinase C-independent staurosporine-sensitive step. *Eur. J. Immunol.*, **21**, 1623-1634.

DE LEIJ L, HELFRICH W, STEIN R AND MATTES MJ. (1994). SCLC-Cluster-2 antibodies detect the pancarcinoma epithelial glycoprotein EGP-2. *Int. J. Cancer*, **8**, (Suppl.) 60-63.

JANSSEN RAJ, BUTER J, STRAATSMA E, HEIJN AA, SLEIJFER D TH, DE VRIES EGE, MULDER NH, THE TH AND DE LEIJ L. (1993). HLA-Dr-expressing CD8 bright cells are only temporarily present in the circulation during subcutaneous recombinant interleukin-2 therapy in renal cell carcinoma patients. *Cancer Immunol. Immunother.*, **36**, 198-204.

JANSSEN RAJ, MULDER NH, THE TH AND DE LEIJ L. (1994*a*). Immunobiological effects of IL-2 *in vivo*. *Cancer Immunol. Immunother.*, **39**, 207-216.

JANSSEN RAJ, HEIJN AA, THE TH, DE LEIJ L. (1994*b*). Poor induction of IL-2R expression on CD8 bright cells in whole blood cell cultures with CD3 MAb. Implications for immunotherapy with low dose CD3 MAb. *Cancer Immunol. Immunother.*, **38**, 53-60.

KROESEN BJ, BUTER J, SLEIJFER D TH, JANSSEN RAJ, VAN DER GRAAF WTA, THE TH, DE LEIJ L AND MULDER NH. (1994). Phase I study of intravenously applied bispecific antibody in renal cell cancer patients receiving subcutaneous interleukin-2. *Br. J. Cancer*, **70**, 652-661.

SLEIJFER D TH, JANSSEN RAJ, BUTER J, DE VRIES EGE, WILLEMSE PHB AND MULDER NH. (1992). Phase II study of subcutaneous interleukin-2 in unselected patients with advanced renal cell cancer on an outpatient basis. *J. Clin. Oncol.*, **10**, 1119-1123.

THOMPSON JA, LEE DJ, LINDGREN CG, BENZ LA, COLLINS C, SHUMAN WP, LEVITT D AND FEFER A. (1989). Influence of schedule of interleukin 2 administration on therapy with interleukin 2 and lymphokine activated killer cells. *Cancer Res.*, **49**, 235-240.

TIBBEN JG, BOERMAN OC, CLAESSENS RAMJ, CORSTENS FHM, VAN DEUREN M, DE MULDER PHM, VAN DER MEER JWM, KEIJSER KGG AND MASSUGER LFAG. (1993). Cytokine release in an ovarian carcinoma patient following intravenous administration of bispecific antibody OC TR F(ab')2. *J. Natl. Cancer Inst.*, **85**, 1003-1004.

VAN OOSTERHOUT YVJM, PREIJERS FWMB, WESSELS HMC AND DE WITTE T. (1992). Cytotoxicity of CD3-ricin A chain immunotoxins in relation to cellular uptake and degradation kinetics. *Cancer Res.*, **52**, 1-5.

WOODLE ES, THISTLETHWAITE JR, GHOBRIAL IA, JOLLIFFE LK, STUART FP AND BLUESTONE JA. (1991). OKT3 F(ab')2 fragments: retention of the immunosuppressive properties of whole antibody with marked reduction in T cell activation and lymphokine release. *Transplantation*, **52**, 354-360.

YOSHINO I, YANO T, MURATA M, ISHIDA T, SUGIMACHI K, KIMURA G AND NOMOTO K. (1991). Cytolytic potential of peripheral blood T-lymphocytes following adoptive immunotherapy and lymphokine-activated killer cells and low-dose interleukin-2. *Cancer Res.*, **51**, 1494-1498.